# Periodic Expansion and Contraction Phenomena in a Pendant Droplet Associated with Marangoni Effect

**DOI:** 10.3390/ma15010239

**Published:** 2021-12-29

**Authors:** Koutaro Onoda, Ben Nanzai

**Affiliations:** Department of Materials and Life Science, Faculty of Science and Technology, Shizuoka Institute of Science and Technology, 2200-2 Toyosawa, Fukuroi 437-8555, Shizuoka, Japan; kirameki-rari-takane@i.softbank.jp

**Keywords:** pendant-drop method, self-oscillation, Marangoni effect, self-organization, interfacial instability

## Abstract

A spontaneous oscillation between the expansion and contraction of a nitrobenzene pendant droplet containing di-(2-ethylhexyl)phosphoric acid (DEHPA) was observed in an aqueous phase under alkaline conditions. We described this phenomenon as the spontaneous oscillation of the oil–water interfacial tension. The oscillation characteristics such as the induction period and the interfacial-tension oscillation frequency were investigated under different temperatures and aqueous phase polarities. The effects of the interfacial tension of the biphasic pendant-droplet, the surface excess of the surfactant molecules, and the amount of nitrobenzene elution from the droplet to the aqueous phase on the oscillation characteristics were investigated. Consequently, the periodic expansion–contraction oscillation mechanism was explained through the adsorption–desorption cycle of DEHPA with respect to the aggregate formation of the inverted micelle of DEHPA. This study was based on a simple vibration phenomenon of interfacial tension, and is extremely important for clarifying the predominant factors that cause fluctuations in the free interface energy, which has been ambiguous.

## 1. Introduction

Various rhythmic oscillating phenomena resulting from the periodic variation of interfacial tension are observable in biphasic systems with clear boundaries between aqueous and oil phase. These periodic oscillations, caused by the surfactant adsorption onto the oil–water liquid interface, are one of the typical self-organization phenomena in systems far from equilibrium. Several investigations on the oscillation phenomenon induced by the Marangoni effect in an oil–water biphasic system have been reported [1,2,3,4,5]. The Marangoni effect is the mass transfer along a liquid–liquid interface caused by the nonequilibrium interfacial tension attributable to the chemical or thermal gradient [2]. Considerable interest exists for understanding the rhythmic phenomena in living organisms and the direct conversion of energy from chemical to mechanical or electrical energy. To date, both numerical simulation and experimental investigation have been conducted to understand the interfacial-tension oscillations [6,7,8]. Shioi et al. observed a traveling wave at an oil–water interface and the oscillation of interfacial tension in a nitrobenzene–water biphasic system with di-(2-ethylhexyl)phosphoric acid (DEHPA) [9]. The variation of interfacial tension was explained by the adsorption–desorption cycle of the surfactant at the oil–water interface owing to the Marangoni effect. Ban et al. reported that a pendant droplet containing a phosphoester surfactant was periodically alternated between expansion and contraction via the oscillations of interfacial tension in buffered aqueous solutions [10]. The mechanisms for the oscillation phenomena of interfacial tension were suggested enough through these previous reports as follows. However, very few discussions for the predominant factor of these oscillation phenomena were reported. Elucidation of the controlling factors is indispensable when considering industrial application, in particular, for liquid/liquid extraction, stability of foams and emulsions, correct measurement of dynamic surface tension, and chemical motors for microfluidic devices. In this study, to clarify the predominant factor of Marangoni effect in the pendant droplet, DEHPA behavior in a pendant droplet with periodic expansion–contraction oscillation will be discussed in detail. The effect of various experimental conditions on the oscillation characteristics, such as induction period and interfacial-tension oscillation frequency, was considered. The interfacial tension in some previous reports was measured using the Wilhelmy method, which is likely to be sensitive to interfacial instability. For the pendant-drop system, the interfacial tension can be ascertained from the image of the droplet using drop-shape analysis via the pendant-drop method.

## 2. Materials and Methods

Wako Pure Chemical Industries, Ltd. (Osaka, Japan) supplied nitrobenzene, DEHPA, sodium carbonate, sodium hydrogen carbonate, methanol, ethanol, and acetone. Aqueous phases with each additive were prepared using Milli-Q water (resistivity of 18.2 MΩ cm at 25 °C). The oil phase was nitrobenzene, and the concentration of surfactant DEHPA was adjusted at 100 mM in the oil phase. The pH of aqueous solution was adjusted to 10.2 by adding a sodium hydrogen carbonate buffer. In this study, organic solvents—such as methanol, ethanol, and acetone—were added in the aqueous phase to vary the polarity of the aqueous phase. The concentration of methanol, ethanol, and acetone in the aqueous solution was adjusted to 0–4 mol L^−1^.

In this study, the experiment on various contents such as measurement of dynamic interfacial tension, measurement of static interfacial tension, estimation of surface excess of DEHPA, and determination of nitrobenzene elution. The oil pendant droplet was formed on the tip of a capillary (15G) in the prescribed aqueous phase stored in a quartz cell (20 × 20 × 30 mm). The aqueous phase was adjusted to a predefined temperature using a temperature control system (4VT; Kyowa Interface Science Co. Ltd., Niiza-City, Japan). The concentration of nitrobenzene dissolved in the aqueous phase was measured after a 2000-s observation of oscillation phenomena using absorption photometry (UV-1280; Shimadzu Corp., Kyoto, Japan). The interfacial tension was measured using a contact angle meter (Drop Master DM-501; Kyowa Interface Science Co. Ltd., Niiza-City, Japan). The dynamic interfacial-tension measurement for the pendant-drop system with DEHPA was conducted under aqueous phase polarities. We also conducted the dynamic interfacial tension measurement under different temperatures with no organic additives. Based on the results of dynamic interfacial tension, the induction period before starting oscillation and the amplitude of interfacial-tension variation were estimated. The static interfacial-tension measurement for the pendant-drop system without DEHPA, defined as ‘initial interfacial tension’, was also conducted under different temperatures and aqueous phase polarities. The surface excess of DEHPA on the oil–water interface was estimated from the interfacial tension variation depending on DEHPA concentration.

## 3. Results and Discussions

### 3.1. Interfacial-Tension Oscillation

**Figure 1:**
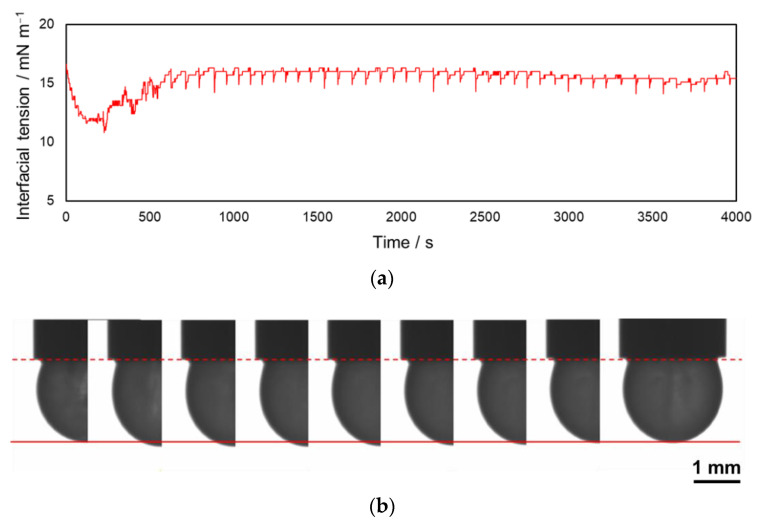
(**a**) Representative variation of interfacial tension synchronized with repetitive cycles of pendant droplet between contraction and expansion. (**b**) Series of images of the contracting and expanding process of the pendant-droplet (33 ms interval).

A droplet hangs from the tip of the capillary when the oil phase is pushed out vertically. From Video S1 presented in Supplementary Materials (5× speed replay; 2 min after the droplet formation), the droplet started to be contracted in a while after pendant-droplet formation (20–30 s in Video S1). Subsequently, the droplet expanded suddenly (47 s in Video S1) and then contracted gradually. Thereafter, the pendant droplet periodically repeated the contraction and expansion cycles. Figure 1 shows the representative variation of interfacial tension. The increase–decrease cycle of the interfacial tension was synchronous with the repetitive contraction–expansion cycle, respectively. This synchronization indicated that the droplet’s oscillating motion depends on the interfacial tension of the pendant-drop biphasic system. In this oscillation phenomenon, the periodic variation of the interfacial tension has characteristics such as the amplitude, frequency, and induction periods. We defined the time from the droplet formation to the first sudden expansion as the induction period of this oscillation phenomenon. Our preliminary investigations demonstrated that the amplitude of interfacial tension’s periodical variation depended on the DEHPA concentration and the pH of the aqueous solution. The pH dependence was discussed in a previous report based on the dissociation of DEHPA [10]. In this study, the amplitude was constant because the DEHPA concentration and pH were kept constant at 100 mmol L^−1^ and 10.2, respectively. For clarifying the predominant factor of these oscillation characteristics such as frequency and induction period, we investigated the effect of some conditions of the biphasic system, such as the polarity and temperature of the aqueous phase.

### 3.2. Effect of Interfacial Tension

**Figure 2:**
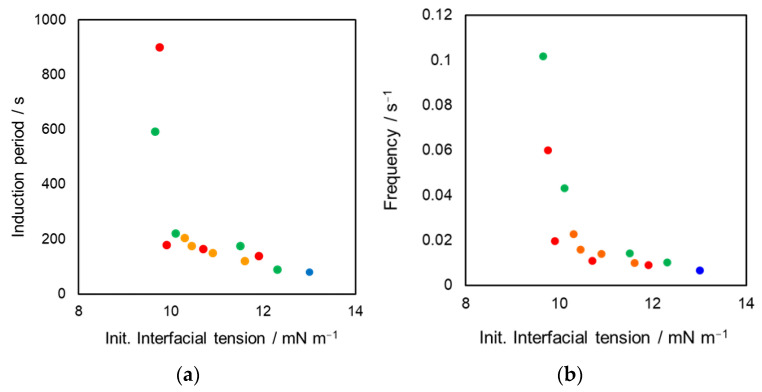
Relation between the oil–water interfacial tension of pendant-drop system without DEHPA and the oscillation characteristics; (**a**) induction period and (**b**) frequency; Each symbol indicates the additive in aqueous phase. ●: no additive, ●: ethanol, ●: methanol, ●: acetone. Each plot was based on duplicate experiments.

**Figure 3:**
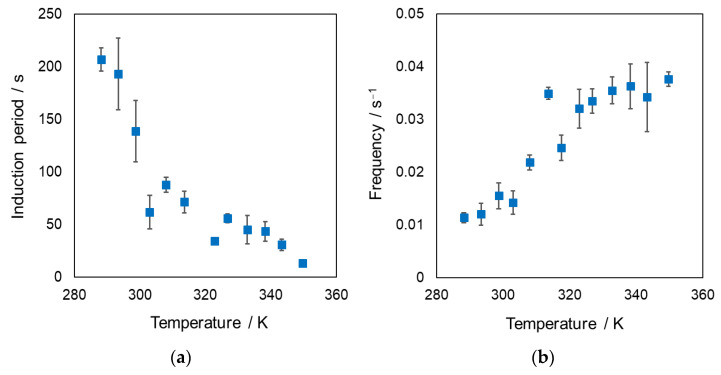
Relation between the temperature of biphasic system and the oscillation characteristics; (**a**) induction period and (**b**) frequency. Only DEHPA was added in these pendant-drop systems, not organic additives. The error bar indicates standard deviation based on triplicate experiments.

In general, the interfacial tension of the oil–water biphasic system strongly depends on the polarity of the aqueous phase. The interfacial tension of the hanging droplet without DEHPA, defined as the initial interfacial tension, was measured to eliminate the complexity of the interfacial instability. Consequently, the obvious involvement of the interfacial tension in the oscillation phenomenon was observed (Figure 2). The reproducibility for these dynamic interfacial tension by two-times experiment and the reproducibility of both characteristics were sufficiently high. The induction period and interfacial-tension oscillation frequency decreased with increasing initial interfacial tension. Furthermore, we assessed the effect of the temperature of the aqueous phase on the oscillation phenomenon. Figure 3 shows the relation between the oscillation characteristics and temperature. The induction period of the oscillation phenomenon decreased and the frequency increased with increasing temperature. These tendencies found in Figure 3 were different from those presented in Figure 2, although the interfacial tension depends on the temperature. Based on the general theory that the interfacial tension may decrease with increasing temperature, the relations between frequency and interfacial tension as well as temperature (Figure 2b and Figure 3b) can be explained via the effect of the initial interfacial tension. By contrast, the induction period may be dependent on a different predominant factor. Therefore, the discussion on the inflection point observed in the relation between induction period and initial interfacial tension as shown in Figure 2a was difficult. A detailed discussion regarding induction period and frequency will be described later section.

### 3.3. Surface Excess Concentration of DEHPA

**Figure 4:**
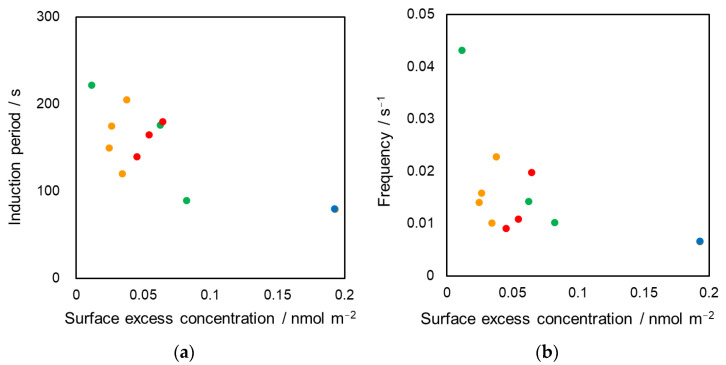
Relation between surface excess, DEHPA concentration on the oil-water interface, and the oscillation characteristics; (**a**) induction period and (**b**) frequency; Each symbol indicates the additive in aqueous phase. ●: no additive, ●: ethanol, ●: methanol, ●: acetone. Each plot was based on duplicate experiments.

Most previous investigations have explained the periodic variation of the interfacial tension based on the repetition between adsorption and desorption of the surfactant [1,3,4,5,6,7,8,9,10,11,12]. Based on this information, the concentration of surfactants adsorbed on the oil–water interface may control the oscillation characteristics. We estimated the surface excess *Γ* (mol m^−2^) of DEHPA in the biphasic system based on the Gibbs adsorption equation as described below.

(1) $$\Gamma =-\frac{1}{RT}\left(\frac{\partial \gamma }{\partial \ln C}\right),$$

where *C* denotes the DEHPA concentration in the pendant-drop phase, *γ* represents the initial interfacial tension, *R* is the gas constant (8.314 J K^−1^ mol^−1^), and *T* denotes the temperature of the biphasic system (room temperature, 298.15 K). These results demonstrated that the DEHPA concentration on the oil–water interface was not the primary factor for controlling the oscillation characteristics (Figure 4). Different tendencies were found in Figure 4 with respect to each pendant-drop system in the aqueous phase with different organic additive. Although the existence of DEHPA was necessary for this oscillation phenomenon, the simple repetition process between DEHPA adsorption and desorption on the interface was unreasonable.

### 3.4. Elution of the Droplet Phase

**Figure 5:**
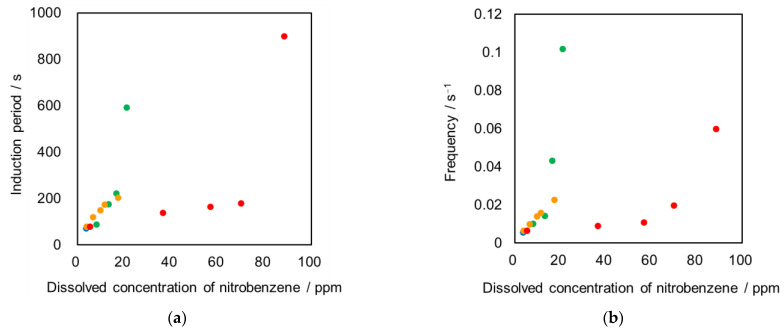
Relation between concentration of nitrobenzene dissolved in aqueous phase and the oscillation characteristics; (**a**) induction period and (**b**) frequency; Each symbol indicates the additive in aqueous phase. ●: no additive, ●: ethanol, ●: methanol, ●: acetone. Each plot was based on duplicate experiments.

In previous reports [5,12,13], the solute concentration in either phase affected the characteristics of the interfacial oscillation phenomenon in the biphasic system because the interfacial instability was induced by the cross-boundary mass transfer. Thus, we assessed the mutual solubility between biphasic systems. We determined that the nitrobenzene concentration dissolved in the aqueous phase affected the polarity of the aqueous phase. The nitrobenzene elution from the droplet to the aqueous phase increased with increasing amounts of organic solvent in the aqueous phase. In each biphasic system with different organic additives, the pendant-drop oscillation frequency increased with increasing solvent concentration in the aqueous phase. This increase was derived from the cross-boundary mass transfer. However, no correlation existed between the concentration of dissolved nitrobenzene and oscillation characteristics (Figure 5). However, the results indicated that the cross-boundary mass transfer did not control this oscillation phenomenon, which should have been a significant factor affecting interfacial instability.

### 3.5. Visual Observation

In the previous reports [9,10,11], the decrease–increase cycle of interfacial tension was explained simply by the adsorption–desorption cycle of disassociated DEHPA ion on an oil–water interface. In fact, the interfacial tension of an oil–water biphasic system should be decreased via DEHPA adsorption owing to the interfacial activity. In this regard, the oscillation phenomenon was not observed in the biphasic system with surfactants other than DEHPA. A previous investigation proposed that the dissociated DEHPA that was adsorbed on the oil–water interface diffused into the aqueous phase [10]. Based on this information, surfactants other than DEHPA should show the same behavior. As shown in Video S1, the first slow contraction of the pendant droplet occurred simultaneously with the oil phase becoming turbid within the pendant droplet. The turbidity was observed only inside the droplet and not in the pendant-drop system without DEHPA. In our study, this process was observed in each pendant-drop system at the beginning of the oscillation phenomenon. An intensive movement of the turbidity was observed synchronously with the convection inside the droplet. This convection flow was expected to be the Marangoni convection that was induced by the gradient of interfacial tension of biphasic pendant-drop system. This convection was observed synchronously with the droplet expansion process in the oscillation cycles. A nonuniform distribution of the turbidity seemed to be uniformized via convection. Consequently, the turbidity may be due to the aggregates of reverse micelle of DEHPA; DEHPA aggregation was previously reported [14,15]. The aggregation comprising dissociated DEHPA was affected by the pH of the aqueous phase because DEHPA dissociated at a pH above its p*K*a of 1.90 in the benzene system [16,17]. This correlated well with the pH dependence results of a previous study [10]. In addition, the involvement of reverse micelle was supported by the results showing that the oscillation phenomenon was observed when the DEHPA concentration above its critical micelle concentration.

### 3.6. Mechanism of the Oscillation Phenomenon

The results obtained in this study enabled us to explain the mechanism of the oscillation phenomena (described below). After the droplet formation, the DEHPA inside the droplet gradually adsorbed on the biphasic interface and then dissociated. This resulted in the initial decrease of the oil–water interfacial tension (before 250 s in Figure 1). Subsequently, the dissociated DEHPA desorbed from the interface into the droplet with the simultaneous formation of inverted micelles. The increased micelles coalesced and formed aggregates inside the droplet. Concomitantly, the biphasic interface tension increased and came close to the oil–water interfacial tension without DEHPA, which was initial interfacial tension defined in this study. The interfacial tension reached a critical state for the oscillation phenomenon (at ~600 s in Figure 1). The time from the droplet formation to this moment was represented as the induction period. The duration of induction period may be affected by the rate of DEHPA desorption associated with the reverse micelle formation. The Marangoni convection occurred owing to the interfacial tension difference between the conditions with and without DEHPA. The interface with and without the dissociated DEHPA had high and low interfacial tensions, respectively. The subsequent DEHPA was supplied immediately from the fresh oil phase in the capillary via the Marangoni convection. The renewed DEHPA adsorption on the oil–water interface induced a precipitous decrease in the interfacial tension. These results correlated well with the numerical simulation reported in a previous investigation [18]. Based on the results obtained in this study, we concluded that the predominant factor of the oscillation phenomena was the difference in the interfacial tension with and without surfactant. We interpreted this to indicate that the intensity of Marangoni convection such as convection velocity was affected by the interfacial tension. Further investigations on the quantification of Marangoni convection in the pendant droplet are required to clarify the relation between Marangoni effect and the interfacial tension in this pendant-drop system. The velocity of Marangoni convection may increase with the initial interfacial tension of pendant droplet because the Marangoni convection would be induced by the gradient of interfacial tension. The oscillation frequency of interfacial tension may decrease with increasing Marangoni convection velocity in the pendant droplet. Based on the suggestion that the DEHPA desorption associated with the reverse micelle formation results in the increasing the interfacial tension, the clearance of droplet surface was induced more efficiently by the Marangoni convection with higher intensity. Subsequently, the droplet interface needed a longer time to be a recurrent condition for the next oscillation.

## 4. Conclusions

The interfacial-tension oscillation of an oil–water interface was observed concurrently with the periodic repetition of the expansion and contraction of a pendant droplet. We found that the expansion occurred simultaneously with the Marangoni convection induced by the interfacial-tension gradient resulting from the DEHPA adsorption–desorption cycle associated with the aggregate formation of inverted micelles. The induction period of oscillation was interpreted as the time until the biphasic interface lost DEHPA via desorption and reached a critical state for inducing the oscillation phenomenon. The frequency of oscillation was controlled by the interfacial tension of the pendant-drop system without DEHPA. These results demonstrate that the periodic Marangoni convection was induced via the molecular behavior of surfactant in the oil–water biphasic system. 

## Data Availability

The data presented in this study are available on request from the corresponding author.

## Supplementary Materials

The following are available online at https://www.mdpi.com/article/10.3390/ma15010239/s1, Video S1: Movie of the oscillating pendant droplet at 2 min after the droplet formation. The movie replays in 5× speed.
